# Smoke, alcohol and drug addiction and female fertility

**DOI:** 10.1186/s12958-020-0567-7

**Published:** 2020-03-12

**Authors:** Cristina de Angelis, Antonio Nardone, Francesco Garifalos, Claudia Pivonello, Andrea Sansone, Alessandro Conforti, Carla Di Dato, Felice Sirico, Carlo Alviggi, Andrea Isidori, Annamaria Colao, Rosario Pivonello

**Affiliations:** 1 I.O.S. & COLEMAN Srl, Naples, Italy; 2 Dipartimento di Medicina Clinica e Chirurgia, Università “Federico II” di Napoli, Via Sergio Pansini 5, 80131 Naples, Italy; 3 FERTISEXCARES Centro di Andrologia, Medicina della Riproduzione e della Sessualità Maschile e Femminile, Università “Federico II” di Napoli, Naples, Italy; 4 Dipartimento di Sanità Pubblica, Università “Federico II” di Napoli, Naples, Italy; 5 Department of Experimental Medicine, Faculty of Medicine and Dentistry, University of Rome “Sapienza”, viale Regina Elena 324, 00162 Roma, Italy; 6 Department of Neuroscience, Reproductive Medicine, Odontostomatology, University of Naples Federico II, Naples, Italy; 7 Cattedra Unesco “Educazione alla salute e allo sviluppo sostenibile”, Università “Federico II” di Napoli, Naples, Italy

**Keywords:** Smoke, Alcohol, Drug, Female Fertility, Reproduction, Ovary, Oviduct, Uterus, PCOS, Endometriosis

## Abstract

**Background:**

Considerable interest has been gathered on the relevant impact of preventable factors, including incorrect lifestyle and unhealthy habits, on female fertility. Smoking, alcohol and addictive drugs consumption represent a major concern, given the broad range of diseases which might be favored or exacerbated by these dependable attitudes. Despite the well-characterized effects of prenatal exposure on pregnancy outcomes and fetus health, a substantial proportion of women of reproductive age is still concerned with these habits. At present, the impact of smoke, alcohol and addictive drugs on women fertility, and, particularly, the specific targets and underlying mechanisms, are still poorly understood or debated, mainly due to the scarcity of well-designed studies, and to numerous biases.

**Objective:**

The current review will provide a comprehensive overview of clinical and experimental studies in humans and animals addressing the impact of smoke, alcohol and addictive drugs on female fertility, by also embracing effects on ovary, oviduct, and uterus, with particular reference to primary endpoints such as ovarian reserve, steroidogenesis, ovulation and menstrual cycle, oviduct function and uterus receptivity and implantation. A brief focus on polycystic ovary syndrome and endometriosis will be also included.

**Methods:**

A Pubmed literature search was performed with selected keywords; articles were individually retrieved by each author. No limitation was set for publication date. Articles in languages other than English were excluded. Additional articles were retrieved from references list of selected manuscripts.

**Results and conclusions:**

Currently, the most consistent evidences of a detrimental effect of smoke, alcohol and addictive drugs on specific domains of the female reproductive function are provided by experimental studies in animals. Overall, clinical studies suggest that smoking is associated to decreased fertility, although causal inference should be further demonstrated. Studies addressing the effect of alcohol consumption on female fertility provide conflicting results, although the majority reported lack of a correlation. Extremely scarce studies investigated the effects of addictive drugs on female fertility, and the specific actions of selected drugs have been difficult to address, due to multidrug consumption.

## Introduction

A significant trend towards a progressive worldwide decline in human fertility over the last five decades has been reported by international literature [1]; therefore, much attention has been raised on identifying environmental and lifestyle modifiable risk factors affecting human reproductive function. Currently, the most commonly accepted definition of infertility is the failure to establish a clinical pregnancy after 12 months of regular unprotected sexual intercourse [2]. Female factors contribute to about 37% of infertility problems, whereas male factors account for about 29%, and combined female and male factors for about 18% of causes; the remaining 16% is due to genetic factors (1%), or to unexplained, or idiopathic, infertility, which is diagnosed in absence of a specific etiological factor (http://old.iss.it/rpma/). Female factors of infertility include ovarian, oviductal, and uterine disorders [3]. A schematic list of causes of female infertility is presented in **Table****1**.

**Table 1:** Causes of female infertility

Ovarian disorders	
Hypothalamus-pituitary-ovary axis dysfunction	
Premature ovarian failure	
Polycystic Ovary Syndrome	
Oviductal disorders	
Pelvic inflammatory disease	
Surgery	
Pelvic tuberculosis	
Uterine disorders	
Benign polyps or tumors (fibroids or myomas)	
Endometriosis scarring or inflammation	
Uterine abnormalities	
Cervical stenosis	
Cervical mucus defects	
Endometriosis	
Idiopathic infertility	

The detrimental role of incorrect lifestyle on human wellbeing has been long time characterized, and unhealthy habits, including smoking, alcohol and recreational or illicit drug (addictive drugs) consumption (use and abuse), represent a primary source of preventable risk factors for several human diseases, as well as a cause of increased morbidity and mortality [4–6]. The effects of smoking, alcohol consumption and drug addiction on female fertility have been heterogeneously investigated. The negative effects of smoking on female fertility have raised much interest in recent years, although most of the evidence is gathered from retrospective studies. Nevertheless, available evidences point out a significant association between both active and passive smoking and reduced female fertility, and between in utero exposure and multiple adverse pregnancy outcomes [7–10], and reduced female descendants fertility at adulthood [3]. These evidences implies that women with fertility disorders, as well as pregnant women, should be advised to stop smoking, or referred to smoking cessation programs [3]. Evidences on the impact of alcohol consumption on female fertility are quite inconsistent, although the majority of studies suggest that moderate alcohol consumption might be unrelated to female fertility; nevertheless, alcohol consumption during pregnancy has been linked to increased risks of adverse pregnancy outcomes and fetal alcohol spectrum disorders, therefore, excessive alcohol intake should be disregarded by women attempting to achieve a pregnancy and pregnant women, and episodes of alcohol intoxication should be avoided [3, 11]. Moreover, both smoking and alcohol consumption might determine epigenetic changes and DNA damage in germ cells, potentially resulting in inherited imprinting and genetic defects, and associated syndromes [12, 13]. Lastly, although poorly investigated, some evidences suggest that use and abuse of addictive drugs might adversely affect female reproductive function, and reduce couple fertility potential; therefore, accurate investigation on addictive drugs consumption should be performed, in women with fertility disorders, and proper counselling should be provided [3].

The current review provides a comprehensive overview on the impact of smoke, alcohol consumption and drug addiction on female fertility, by encompassing the effects on fertility outcomes, and on the ovary, oviduct, and uterus physiology, with particular reference to primary endpoints such as ovarian reserve, ovarian steroidogenesis, ovulation and menstrual cycle, oviduct function, and uterus receptivity and implantation; the relationship between smoke, alcohol consumption and drug addiction and pregnancy outcomes or neonatal health is tangential to the scope of this review and is not covered by the present dissertation. A brief focus on polycystic ovary syndrome (PCOS) and endometriosis, as major causes of female infertility, is also included.

## Search strategies and data extraction

For the present narrative review articles were individually retrieved by each author by literature search in PubMed (MEDLINE) using each of the following terms: “smoke”, “smoking”, “smoker”, “cigarette”, “tobacco”, “alcohol”, “alcoholic”, “beverage”, “drug”, “addictive drug”, “addiction”, “recreational drug”, “marijuana”, “cocaine”, “methamphetamine”, “heroin” combined to each of the following terms: “female fertility”, “female infertility”, “reproduction”, “reproductive function”, “ovary”, “ovarian reserve”, “steroidogenesis”, “oviduct”, “uterus”, “ectopic pregnancy”, “implantation”, “PCOS”, “polycystic ovary syndrome”, “endometriosis”. No limit was set concerning date of publication. Additional references included in the retrieved articles, meta-analyses and reviews were included. Articles in languages other than English were excluded. Articles mostly focusing on pregnancy outcomes and fetal health were excluded, with the exception of data on pre-pregnancy outcomes, which were considered for the present review. In case of duplication of data, the most comprehensive or updated articles were selected, same criterion applied for review articles.

## Smoke and female fertility

**Fig. 1:**
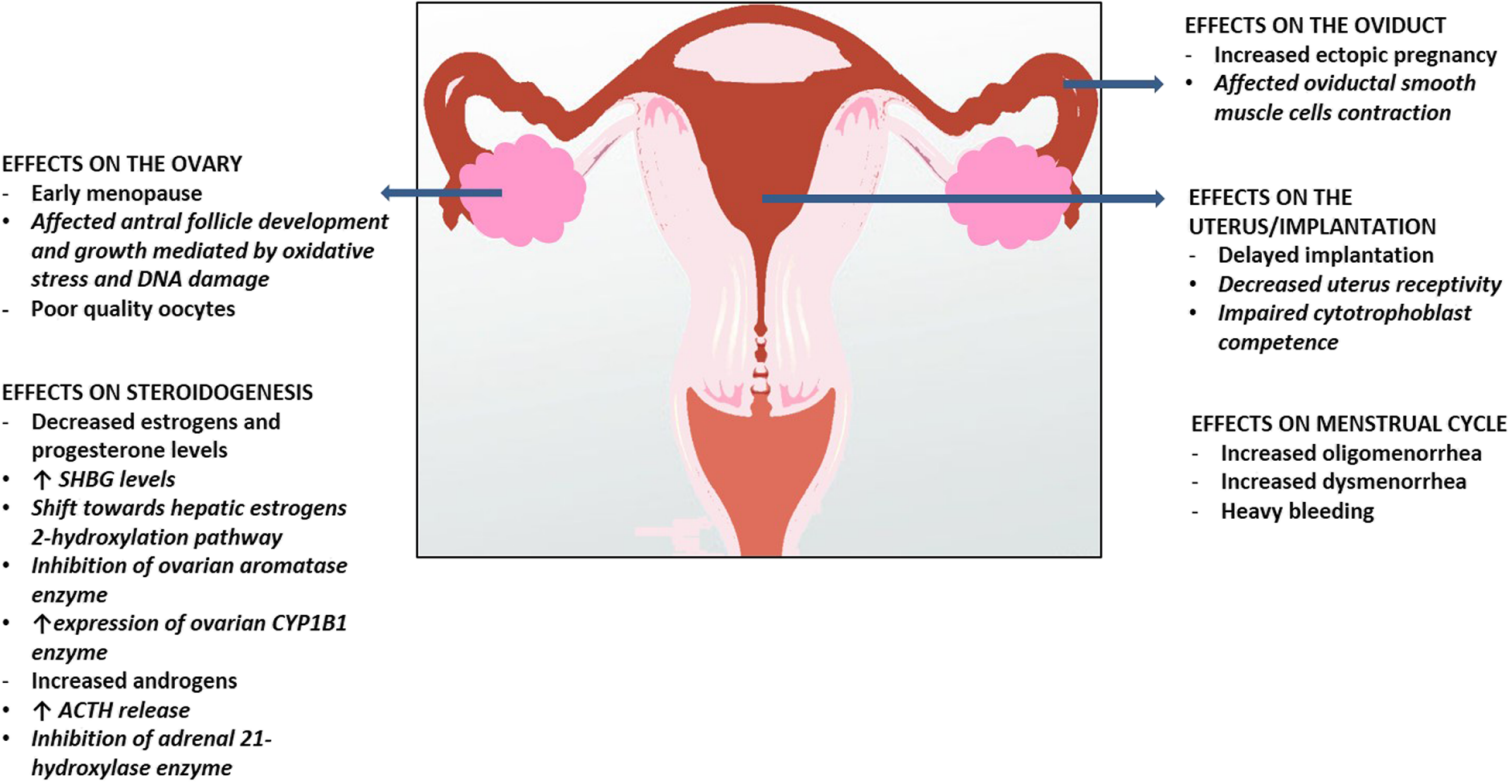
Graphical summary of the main effects of smoking on the reproductive function in women. Smoking affects nearly all domains of the female reproductive function. Smoking is associated to early menopause and reduced levels of ovarian reserve markers, mediated by an impairment of antral follicle development and growth, due to supportive granulosa cells-directed oxidative stress and DNA damage, resulting in cytotoxicity and production of poor quality oocytes. Smoking is associated to lower estrogens and progesterone and higher androgens levels, effects mediated by both ovarian and extra-ovarian actions, including: increased levels of sex hormone binding globulin (SHBG); increased hepatic production of estrogens metabolites with minimal estrogenic activity by pushing the estrogens 2-hydrohylation pathway; inhibition of aromatase enzyme; increased expression of ovarian CYP1B1 enzyme; increased levels of adrenocorticotropic hormone (ACTH); inhibition of adrenal 21-hydrohylase enzyme. Smoking is associated to an increased risk of ectopic pregnancy, mediated by affected oviductal smooth muscle contractility, to delayed implantation, mediated by reduced endometrium receptivity and cytotrophoblast proliferation, migration and invasion, and to an increased risk of oligomenorrhea, dysmenorrhea and menstrual symptoms, although apparently not determined by ovulatory dysfunction

Smoking represents a worldwide health concern, and has been consistently demonstrated to play a role in a vast number of human diseases [14], including reproductive disorders [3, 15]. Despite the substantial evidence on its deleterious effects on female reproduction, and remarkable campaigns against smoking sustained by health-care providers, still 175 millions of women aged 15 or older worldwide are current, daily or non-daily, smokers [16, 17]. Robust evidences suggest that smoking affects several aspects of the female reproductive function, and, therefore, natural female fertility, by exerting multiple differential effects on several targets, including the ovary, oviduct, and uterus [18–20]. Moreover, smoking has been shown to decrease the outcome of assisted reproductive technologies (ART) [21]. Cigarette smoke contains about 4000 substances belonging to a variety of chemical classes, including polycyclic aromatic hydrocarbons, heavy metals, and alkaloids, which are all compounds displaying reproductive toxicity [20, 22–24]; therefore, cigarette smoke is a complex mixture of compounds potentially exerting composite effects on different targets within the reproductive system. The specific effects of the individual cigarette smoke constituents on female reproductive function have been extensively described elsewhere [18–20], and won’t be further reported by the current review, which is an appraisal of the impact of lifestyle, including smoking habits on female fertility; the effects of smoking on female reproductive function and fertility have been investigated in clinical observational studies in humans, in experimental studies on human tissues and cells, and in animal models. A graphical summary of the main effects of smoking on the reproductive function in women is depicted in **Fig.****1****.**

### Female fertility

Several observational studies evaluated the impact of smoking on female natural fertility or ART outcomes; the majority of studies supported the evidence of an increased prevalence of infertility or subfertility in smokers. A metanalysis of studies pointed out a significant association between smoking and infertility, by reporting, overall, a 60% increase in the risk of infertility [25]. Noteworthy, these results were consistent across studies with different designs [25, 26], although definitive inference of a causal association cannot be robustly supported; indeed, only one study within the metanalysis had a prospective design, and failed to detect any significant difference in fertility between smokers and non-smokers, after adjusting for potential confounders [27], and only few studies demonstrated a dose-response relationship [28–31]. Nevertheless, the evidence that past smokers had no different odds for infertility compared to non-smokers might suggest the hypothesis that current, rather than past smoking might affect female fertility [32, 33]. Limitations of the metanalysis included clinical heterogeneity, mainly due to different definitions or types of infertility and different age at diagnosis, the observational and mostly retrospective nature of study designs, the misclassification of smoking habits due to self-reported exposure and to women in the control group quitting smoking because of pregnancy, and the selection bias due to the evidence that a minority of infertile women seek for medical treatment for infertility or to differences in the demographic characteristics of women seeking medical treatment for infertility compared to women not referring to medical services. A prospective observational cohort study evaluating the trans-generational effects of smoking on female fertility demonstrated that odds for fertility at adulthood were similarly reduced in non-smokers with in utero exposure to maternal smoke and in smokers without in utero exposure to maternal smoke, compared to non-smokers without in utero exposure to maternal smoke [34]; these findings suggested that smoking during pregnancy does have an effect on female offspring fertility, which is in line with a previous report using a data set from a prospective study [35], and was confirmed by a more recent, large, cohort study, reporting a higher proportion of time to pregnancy >12 months in women with in utero exposure [36]. Observational studies on the relationship between smoking and fertility in women undergoing ART are more controversial; indeed, although inconsistent changes were reported on number of oocytes retrieved and fertilization rates, significantly lower and higher odds were detected for clinical pregnancy *per* cycle, and ectopic pregnancy, respectively, in smokers, by the majority of studies [8, 18, 21, 25, 37], and passive smoking was found to be as damaging as active smoking, concerning implantation and pregnancy rate [38].

In conclusion, the majority of studies suggest that natural fertility is decreased in current smokers and women with prenatal exposure to parental smoke, whereas relatively scant studies report a controversial relationship between smoking and ART outcomes.

### Ovarian reserve

Observational and experimental studies in humans and animals suggest that smoking might affect folliculogenesis and oogenesis, by means of direct ovotoxicity and central actions on the hypothalamus-pituitary-ovary (HPO) axis. An association between smoking and elevated levels of follicle stimulating hormone (FSH) and changes in anti-müllerian hormone (AMH) levels, a marker of ovarian reserve, or antral follicle count (AFC) has been highlighted in some human studies, suggesting a role in ovarian aging; nevertheless, whereas experimental studies in animals consistently suggest an effect on primordial follicle pool and ovarian reserve, human studies are limited and heterogeneous in design, and further validation is needed. Scarce literature exists as concerns the relationship between smoking and premature ovarian failure (POF).

Current, but not past, smoking, has been shown to be the most consistent and established independent risk factor for younger age at natural menopause [39, 40], with an estimated impact of about one year [40]; moreover, although no association was found with serum levels of AMH [41], current smokers were found to have lower age-specific AMH percentiles [42], and a more rapid decline in AMH slope, relative to age at final menstrual period [43]. Studies in late-reproductive-age and peri-menopausal women demonstrated that current smokers had significantly reduced levels of AMH, whereas past or passive smoking had no effect [44]; moreover, FSH levels were higher in current and passive smokers, whereas past smoking had no effect [45]. These results suggested a possible direct effect of current smoking on antral, but not primordial follicles atresia, in this subset of women. Indeed, the depletion of growing follicles could lead to a decline in the level of their secreted markers AMH and inhibin B, with an increase in FSH levels; unaffected primordial follicles could, on the other hand, replenish growing follicles pool at smoking cessation, by inducing the normalization of AMH, inhibin B, and FSH levels, as observed in past smokers [44]. Studies on pre-menopausal women demonstrated that smoking was not associated to non-growing follicle (NGF) (primordial, intermediate, and primary follicles) count [46], and, in line with results on peri-menopausal women, was associated to increased FSH levels, but not to AMH levels [47], suggesting that a direct role in antral follicle atresia might be questionable. Studies on women enrolled in ART are controversial in this regards; indeed, no difference in AMH levels and in small AFC was reported in smokers by one study [48], although different studies showed either decreased AMH levels and altered follicles size repartition in AFC [49], or decreased follicular fluid AMH levels [50], highlighting the need of further dedicated studies. Experimental studies on ovarian cells retrieved from women undergoing ART demonstrated that a major ovotoxic action of smoking is oxidative stress and DNA damage in granulosa cells, which might interfere with cell maturation, binding of gonadotropins to their receptors, and oocyte fertilizing capacity [18, 51]. Indeed, in smokers, a significantly increased expression of antioxidant enzymes, suggestive of smoke-induced oxidative stress, was detected in granulosa cells [52], and a significant increase in DNA damage was reported in oocyte cumulus cells [53]; moreover, morphological assessment of oocytes collected from women undergoing ART demonstrated an increased thickness of zona pellucida [54], and an increased frequency of meiotic immature diploid oocytes, probably resulting from prevention of first polar body extrusion [55]. Fetal development is a crucial phase during which germ cells complete proliferation, initiate meiosis and form the lifetime stock of primordial follicles; few studies investigated the reproductive trans-generational effects of parental smoking. Studies on human fetal ovaries from legal abortions demonstrated that maternal smoking deregulated several genes involved in ovarian developmental signalling [56], and significantly decreased the number of somatic cells, whereas the number of oogonia only displayed a trend towards reduction [57]; since proper oocyte development and maturation require that oogonia invading the developing ovary become enclosed by somatic cells in a primordial follicle, the results of this study suggested that long-term ovarian function in female offspring might be affected. Nevertheless, no association between maternal smoking during pregnancy and ovarian volume, AFC, and FSH, AMH and inhibin B levels, assessed in female descendants at adolescence, was reported by prospective cohort studies [58, 59], whereas paternal smoking was associated to decreased AMH levels [60]. Lastly, few and dated studies evaluating risk factors for POF in different populations reported controversial results concerning the impact of smoking; one case-control study investigating pre-menopausal risk factors demonstrated that smoking was associated to an increased risk of idiopathic POF [61], whereas different studies failed to find any association [62, 63]. Taken together, these evidences suggest that the association of smoking with earlier age at natural menopause and impaired, although controversial, levels of ovarian reserve markers, might involve processes other than accelerated follicular atresia and depletion of ovarian primordial follicle pool, processes which might include either impaired antral follicle development, or HPO axis function, namely, increased FSH production by the pituitary.

In such a complex, dynamic, and tightly regulated setting, the cross-sectional design of most studies dampens the possibility of differentiating between the two potential mechanisms and affects the reliability of hypothesis on eventual causal relationships; indeed, theoretically, active smoking could firstly determine a rise in FSH levels, which would subsequently lead to follicle impairment and decline in AMH levels, or the exact opposite sequence of events. Moreover, time-frame of exposure and of endpoints assessment might represent a crucial point, given the differential susceptibility of the ovary and the diverse developmental stages occurring from fetal life onwards. Experimental studies on adult and prenatal smoke exposure in animals do not support the hypothesis of a primordial follicle-sparing action. An in vivo study in mice subjected to direct nasal exposure demonstrated primordial follicle depletion, antral follicle oocyte atresia, and increased oxidative stress, resulting in decreased fertility potential [64]. Underlying mechanism of ovotoxicity included deregulation of genes associated with detoxification, inflammation, premature primordial follicles activation and immune cell-mediated apoptosis [64]. The effects of smoke exposure on ovarian follicle growth were found to be persistent even after exposure cessation [65]; in vivo studies in rats [66] and mice [67, 68] demonstrated that smoke exposure decreased the number and maturation of ovarian follicles [66], reduced oocytes quality, characterized by increased thickness of zona pellucida [68], reduced oocyte diameter and induced a misshapen first polar body [67], and interfered with oocyte chromosome congression or meiotic spindle shape [68]. Experimental studies in animals subjected to prenatal exposure to smoke consistently suggested a detrimental effect of smoking on primordial follicles. A study on rats exposed from proestrous phase of estrous cycle throughout pregnancy demonstrated significantly increased DNA damage and apoptosis in granulosa cells and ovarian surface epithelium, and significantly reduced ovarian follicle counts, including primordial and growing follicles, in female offspring [69]. Consistently, studies in prenatally exposed mice confirmed abnormal cell proliferation and increased apoptosis in granulosa cells and oocytes, and a significantly reduced ovarian follicle count, at neonatal age [70]; moreover, at adulthood, abnormal cell proliferation and reduced follicle counts, but not apoptosis, persisted, and increased oxidative stress and aberrant metaphase II spindle, markers of poor quality, were detected in oocytes [70].

In conclusion, observational studies suggest that smoking might affect ovarian aging and ovarian reserve markers, although the relationship between smoking and POF is controversial; moreover, experimental studies in humans suggest that an impairment of antral follicle development and growth due to supportive granulosa cells-directed toxicity, rather than primordial follicle pool depletion, might mediate these effects.

### Steroidogenesis

Observational studies highlighted that smokers are characterized by lower estrogens and progesterone [71, 72], and higher androgens [73–75] levels in the circulating blood, as well as lower estrogens levels and an increased androgens/estrogens ratio in the follicular fluids [76]; these evidences are suggestive of impaired steroidogenesis, or steroid hormones metabolism, occurring at both extra-ovarian and ovarian level. A large body of evidences from observational studies suggest that smoking exerts multiple anti-estrogenic actions, by interfering with estrogen biosynthesis, bioavailability, catabolism, and clearance; the potential mechanisms underlying these actions include: the increase of sex hormone binding globulin (SHBG) levels resulting in lower levels of biologically active free estrogens [77]; the increase in production of estrogen metabolites with minimal estrogenic activity [78] which shifts away from the more estrogenically potent 16a-hydroxylation towards the 2-hydroxylation pathway, with production of metabolites which are rapidly cleared from the circulation [79]; and the potential increased estrogen hepatic metabolism [80], the latter being hypothesized by reduced estrogens levels in a cohort of post-menopausal women under hormone-replacement therapy. Consistently, smoke-related mechanisms have been also shown to jeopardize estrogen replacement therapy efficacy [81]. The pro-androgenic actions of smoking might depend on the increased adrenocorticotropic hormone (ACTH) release from the pituitary, which subsequently increases adrenal androgens secretion [82], and the inhibition of adrenal 21-hydroxylase enzyme [83]. As far as ovarian actions are concerned, experimental in vitro studies on human granulosa cells [84] and luteinized granulosa cells [85], demonstrated that treatment with cigarette smoke extracts significantly inhibited the conversion of androstenedione to estradiol, by a dose-dependent inhibition of aromatase activity [84], and significantly decreased both progesterone and estradiol levels, by a significant dose-dependent increase in the expression of CYP1B1 enzyme [85]. The relationship between smoking and steroidogenesis in relation to different phases of the menstrual cycle was investigated in few and conflicting observational studies. A prospective case-control study evaluated plasma levels of luteinizing hormone (LH), FSH, estradiol, androgens, and SHBG, salivary levels of progesterone, and urinary levels of estradiol, estriol and estrone, throughout the late follicular and luteal phase of the menstrual cycle; follow up covered one entire menstrual cycle [86]. The study highlighted that smoking did not consistently suppress LH pulsatility, and had no effect on steroid hormones levels and estradiol/estrone ratio [86]. Conversely, a more recent prospective case-control study with a two menstrual cycles follow up reported significantly higher levels of FSH and LH in the early follicular phase and at menses, respectively, in smokers, compared to non-smokers, although no significant differences were observed in longitudinal models for estradiol, progesterone, or SHBG [87]. Discrepancies between studies might be attributable to very small sample size, and to the reliance upon self-reported smoking data. Lastly, a larger population-based study evaluated the relationship between smoking and follicular phase serum levels of four different estrogens, namely, estradiol, estriol, estrone, and 16-hydroxyestrone, as well as progesterone, by differentiating active from passive smoking [72], demonstrating that, overall, smokers tended to have lower serum estrogens and progesterone levels, compared to non-smokers; in particular, estrone and 16-hydroxyestrone exhibited an inverse dose-dependent relationship with smoking status, with active smokers displaying lower serum levels, compared to passive smokers, and passive smokers displaying lower serum levels, compared to non-smokers, although significance was reached only for 16-hydroxyestrone [72], whereas, for progesterone levels, significant difference was found only between passive smokers and non-smokers, whereas active and passive smokers had only slightly different progesterone levels [72]. Limitation of the study included the cross-sectional design with a one-point measurement of serum hormone levels, and certain discrepancies between self-reported smoking status and serum levels of cotinine, an alkaloid found in tobacco and the predominant metabolite of nicotine used as a biomarker of smoke exposure, which might reflect some misclassification of participants. Larger and better-designed prospective studies and additional experimental studies are needed to definitely address the effects of smoking on steroidogenesis, in relation to menstrual cycle and female fertility.

In conclusion, smoking is associated to changes in the female endocrine profile, mediated by both ovarian and extra-ovarian directed actions, which determine the onset of a hormonal milieu characterized by lower estrogens and progesterone and higher androgens levels.

### Ovulation and menstrual cycle

The relationship between smoking, ovulatory dysfunction, and menstrual cycle disorders or menstrual symptoms has been largely investigated in humans; the majority of observational studies suggest that smokers are at higher risk of a range of menstrual problems, including oligomenorrhea, heavy bleeding, and dysmenorrhea. A large retrospective study on women enrolled in a weight reduction program demonstrated that heavy smokers had increased risk of oligomenorrhea, with a greater risk in women approaching menopause than for younger women [88]. A prospective cohort study demonstrated that smokers have a decreased duration of bleeding, and an increased daily amount of bleeding, in particular within the first two days of menses, whereas no difference in cycle length was found; these changes were dose-dependent, with heavy smokers reporting the more severe effects [89]. Conversely, a different prospective cohort study reported that heavy smoking was associated with an increased risk of shorter menstrual cycles (less than 25 days), most likely due to shortening of follicular phase; moreover, no significant association was found with the risk of anovulation, although odds were increased [90]. A cross-sectional study on women interviewed about specific menstrual symptoms demonstrated that both current and past-smokers had an increased risk of pre-menstrual tension, irregular periods, and heavy periods, with heavy smokers and women with younger age at starting smoking being at the highest risk [91]. The relationship between smoking and the prevalence of dysmenorrhea has been largely investigated. The majority of studies reported an increased risk in smokers [92–94], and a dose-dependent and time-dependent relationship with both active and passive smoking [95, 96]; moreover, severe grade dysmenorrhea was found to be more likely to occur in smokers, and the severity of symptoms increased with the number of cigarettes smoked *per* day [94]. In addition, few longitudinal studies showed that smoking was associated to an increased probability of having a longer duration of severe pain within the menstrual cycle [89, 97], and to moderately increased risk of suffering from chronic dysmenorrhea [98]. Only few studies failed to find an association between smoking and the risk of dysmenorrhea [99–101].

In conclusion, smoking seems not to be associated to ovulatory dysfunction, despite an increased risk of oligomenorrhea and several menstrual symptoms.

### Oviduct function

Ectopic pregnancy is thought to be determined by embryo retention within the oviduct, due to oviductal dysfunction. Migration throughout the oviduct of oocyte cumulus complex and embryo is required for proper fertilization, and transfer of the embryo to the uterus, respectively, and for subsequent implantation, and involves a concerted ensemble of oviductal functions which comprises adhesion of oocyte cumulus complex to oviductal epithelium, ciliary activity of epithelial cells, which generates oviductal fluid movement, which in turn promotes the transfer of the embryo to the uterus, and oviductal smooth muscle contraction, which also contributes to embryo transport [102]; each of these functions might be a specific target of cigarette smoke, therefore contributing to the occurrence of ectopic pregnancy. Large case-control and population-based studies reported that both active and passive smoking are associated to significantly higher odds for ectopic pregnancy [103–106], with significantly increased odds for one or more ectopic pregnancies in active smokers during reproductive age, and in non-smokers participants with the highest levels of lifetime passive exposure, compared to non-smokers with no passive exposure [103]. Moreover, smoking was identified as a major risk factor for ectopic pregnancy [104], although this finding should be interpreted with caution, given that smoking has also been found to be associated with other risk factors for ectopic pregnancy, such as tendency to have multiple sexual partners and pelvic inflammatory disease [107]. Experimental studies investigating the mechanisms beneath the association of smoking with ectopic pregnancy mainly focused on oviductal smooth muscle contraction, epithelium ciliary beat frequency, oocyte adhesion to oviductal ciliated epithelium, and oocyte pick-up rate. One study on women of child-bearing age demonstrated that oviductal motility is compromised by smoke inhalation, as assessed by Rubin test [108]. Results from an experimental study on oviducts collected from women undergoing hysterectomy demonstrated that oviducts from smokers had a significantly higher expression of PROKR1 [109], a G-protein-coupled receptor involved in smooth muscle contractility [110], which was also found to be overexpressed in women with ectopic pregnancy [109]. Experimental studies in hamsters further corroborate the detrimental effect of smoking on oviductal smooth muscle cells contraction. In particular, inhalation of either mainstream or side-stream smoke raising the serum level of cotinine to concentrations mimicking those reached in active or passive smokers, respectively, significantly inhibited muscle contraction of the ampulla, and slowed embryo transport throughout the oviduct, an effect which was not completely reversed upon inhalation withdrawal [111], and decreased the ratio of ciliated to secretory cells in the ampulla [112]. In addition, experimental studies investigating the effects of smoke extracts from several brands and types of cigarette, demonstrated a dose-dependent inhibition of oviductal ciliary beat frequency and muscle contraction (by 30–98%), and inhibition of oocyte pick-up rate (by 50–80% )[19]. Interestingly, side-stream smoke extract slightly increased or had no effect on oviductal ciliary beat frequency, although it was still able to reduce oocyte pick-up rate [19], suggesting that different factors might be responsible for smoke-induced decrease in oocyte pick up. Indeed, an experimental in vitro study using the hamster infundibular model, demonstrated that both mainstream and side-stream smoke extracts increased adhesion of the oocyte cumulus complex to the tips of the oviductal cilia, therefore affecting oocytes pick-up rate [113], an effect which also contribute to delay or inhibit the transport throughout the oviduct, and potentially increases the chance for ectopic implantation.

In conclusion, significantly higher odds for ectopic pregnancy were found in smokers, which is probably mediated by detrimental actions on oviductal smooth muscle contraction.

### Uterus receptivity and implantation

Uterine implantation involves a tight interaction between a receptive endometrium and a competent blastocyst, and a properly and precisely timed remodelling of endometrium is required prior to embryo arrival within the uterus [114]; priming of endometrium might be affected by an estrogen-deficient environment, or poor corpus luteum development, which determine low endometrial receptivity and implantation failure [114]. Moreover, trophoblast gene expression might have a significant role in the interaction with endometrial lining, and damaged trophoblast may not succeed in implantation [114]. Both endometrium receptivity and trophoblast gene expression have been shown to be affected by smoking, and current smoking was found to be associated to delayed implantation [115]. A retrospective study sought to determine the specific effect of smoking on uterus receptivity, by excluding confounders, such as the effect of smoking on ovary, oocyte quantity and quality, embryo quality, and oviductal transport [116]. To this aim, the study investigated implantation rate and pregnancy rate, after embryo transfer in oocyte donor cycles, by restricting the analysis to oocyte donors with known no-smoking or light smoking status (0-10 cigarettes *per* day), and non-smoker husbands, to further exclude the effect of passive smoking; the results of the study demonstrated that both implantation and pregnancy rate were reduced in heavy smoker recipients, compared to the joint non-smoker and light smoker group of recipients, although only changes in pregnancy rate were significant [116]. Consistently, experimental studies demonstrated that human endometrial tissue from smokers had a significantly decreased expression of uterine receptivity markers, and the in vitro treatment with cigarette smoke extract of endometrial stromal cells significantly reduced the expression of these markers [117], and treatment with smoke solution significantly and dose-dependently decreased endometrial epithelial cell proliferation, by interfering with nitric oxide (NO)-mediated pathways [118]. Lastly, human uterine endothelial cells incubated with smoke-conditioned medium displayed aberrant expression and localization of molecules involved in endothelial cell adhesion, and had impaired morphology and migration potential, results which suggested an effect of smoke on endometrial angiogenesis [119]. Experimental studies on human tissues suggested that maternal smoking also affects trophoblast proliferation, migration and invasion, effects which might justify the reduced implantation rate observed in smokers. In particular, experimental studies on first-trimester chorionic villi demonstrated that, in samples retrieved from mothers who smoked more than 20 cigarettes *per* day, the number of Ki67-positive cytotrophoblasts was dramatically decreased, hence indicating that maternal smoking reduced mitotic index and progression of cell cycle [120]; moreover, floating villi displayed focal defects including absence of cytotrophoblast stem cells, and thinner syncytium [120]. These results suggested that maternal smoking induced a premature depletion of the cytotrophoblast stem cell population [120]. A different study demonstrated that maternal smoking dose-dependently down-regulated the cytotrophoblast expression of selectin system, comprising cell adhesion molecules involved in the connection between the fetal portion of the placenta and the uterus, which is required for proper migration and invasion of the uterine cavity, and, therefore, implantation [121].

In conclusion, smoking affects both endometrium receptivity and cytotrophoblast proliferation, migration and invasion, therefore determining delayed uterine implantation.

## Alcohol and female fertility

**Fig. 2:**
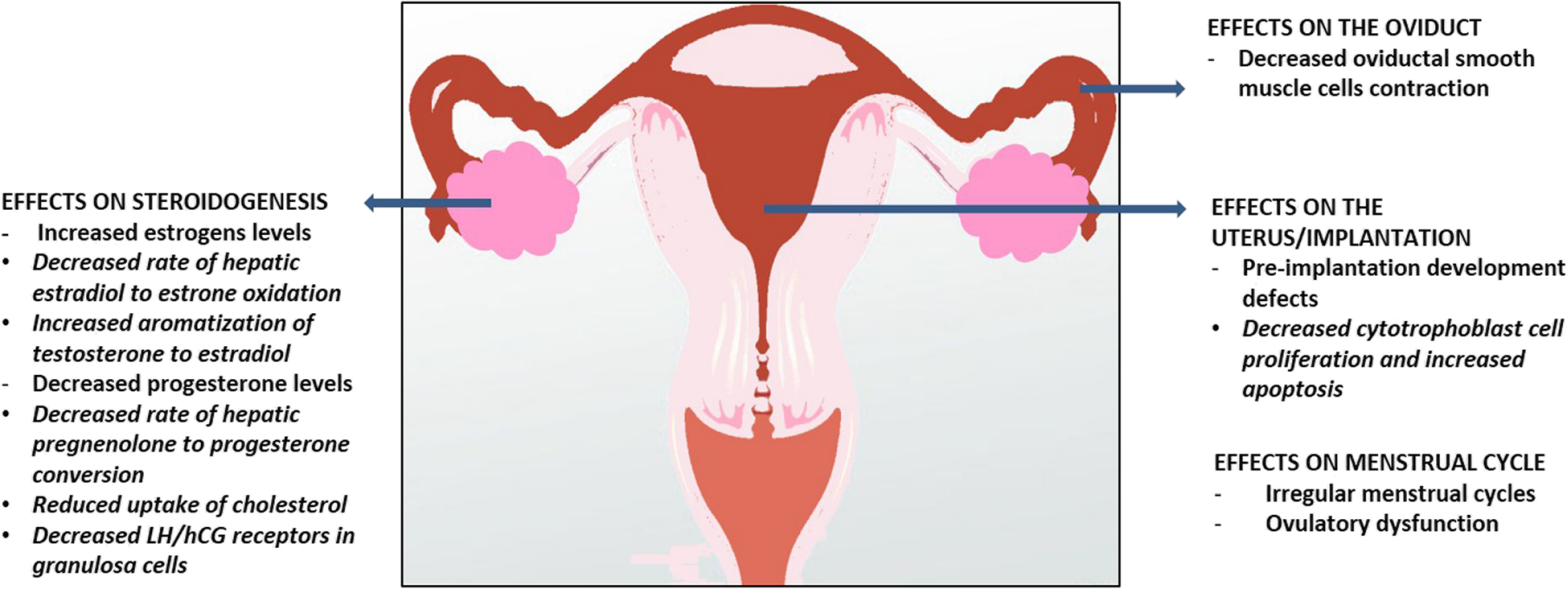
Graphical summary of the main effects of alcohol consumption on the reproductive function in women. Alcohol consumption is associated to higher estrogens and lower progesterone levels, effects mediated by both ovarian and extra-ovarian actions, including: decreased hepatic oxidation of estradiol to estrone; increase of aromatase activity; decreased hepatic conversion of pregnenolone to progesterone; reduced cholesterol uptake; decreased expression of luteinizing hormone (LH) human chorionic gonadotropin (hCG) receptors expression in granulosa cells. Alcohol consumption is associated to reduced oviductal smooth muscle cells contractility, although apparently not resulting in increased ectopic pregnancy rate, and to reduced cytotrophoblast proliferation, and increased cytotrophoblast apoptosis. Alcohol consumption is associated to irregular menstrual cycles and ovulatory dysfunction

Alcohol abuse, conceived as either acute episodes of binge drinking, or steady drinking, intended as daily chronic alcoholism, represents a threat to human health, by increasing the risk of several harmful conditions, such as injuries, violence, poisoning, and of severe chronic diseases [5]; moreover, alcohol use and abuse during pregnancy exerts well known detrimental effects on the fetus, including miscarriage, stillbirth or fetal alcohol spectrum disorders [3, 122]. The effects of alcohol consumption on the female reproductive function and fertility are less described and presently still pose more questions than answers. Most studies compared the differential effect of different rates (low, moderate, high) of alcohol consumption, whereas others focused on a specific level of intake, by mainly addressing the effects of moderate intake of alcohol; nevertheless, heterogeneity of classification has to be carefully accounted, which is based on highly discordant cut-off values for alcohol intake categories, and on the choice of different units for intake measurement, with only a minority of studies routinely applying the “gram”, and a majority of studies quoting the more elastic variable of “drink”, therefore making it challenging to determine a standardized threshold for consumption extent and frequency. Moreover, most studies did not distinguish between binge and steady drinking, or addressed the occurrence of hangover, which might be relevant if large loads of alcohol are consumed during the fertile timeframe of the menstrual cycle. Lastly, the effects of alcoholism on fertility should be considered with caution, since chronic liver disease *per se* plays a relevant role on the female reproductive function. A graphical summary of the main effects of alcohol on the reproductive function in women is depicted in **Fig.****2****.**

### Female fertility

Observational studies evaluating the impact of alcohol consumption on female natural fertility provide conflicting results, although the majority of studies reported lack of a correlation, whereas studies on assisted reproduction are more consistent, and suggest a detrimental effect of alcohol on ART outcomes; only few studies specifically investigated fertility outcomes in alcohol abusers. A large prospective cohort study evaluated the relationship between alcohol consumption and hospitalization for pregnancy outcomes and infertility examinations, by reporting an increased risk of undergoing to infertility examination with high (more than 140 g *per* week) alcohol intake [123]. A major bias of the study included the lack of information on smoking habits, together with the fact that estimates on alcohol consumption was in most cases collected several years before conception. Consistently with these results, however, a retrospective population-based study reported increased time to pregnancy in women with high (more than 7 drinks *per* week) alcohol consumption [124]. A different prospective cohort study on couples seeking first time pregnancy demonstrated an inverse dose-dependent relationship between alcohol consumption and odds of fecundability [125]. Moreover, a reduction of more than 50% in the conception rate was reported in a menstrual cycle during which participants had consumed alcohol (1-90 g *per* week) [126]. Lastly, one case-control study addressing the impact of alcohol consumption on female infertility, as stratified by cause of infertility, demonstrated that both moderate (0-1 drinks *per* day) and high (more than 1 drink *per* day) alcohol consumption significantly increased the risk of ovulatory infertility [127]. By contrast, numerous studies highlighted that alcohol consumption was unrelated to female infertility, by reporting consistent results across different study designs and assessed endpoints. A large population-based cohort study suggested that consumption of any amount of alcohol (0-7+ drinks *per* week) was unrelated to infertility among younger women, although it was a significant predictor for infertility among women above age 30 [128]. A different study based on data retrieved from a case-control study on risk factors for spontaneous abortion, found no differences in the occurrence of difficulty in conception, defined as taking two or more years to conceive or receiving medical treatment for infertility, in women with moderate (1-3 drinks *per* day) alcohol consumption [129]. A more recent prospective cohort study focusing on ovulatory infertility, further confirmed the lack of a relationship between alcohol consumption and the risk of infertility, a result which persisted in sensitivity analyses accounting for different types of alcoholic beverages, including wine, beer and spirits [130]. A population-based case-control study on fertile women and women with any cause of primary infertility, found no significant changes in the average time to conception across levels of alcohol consumption (0-5+ drinks *per* week) [131]; these findings were also confirmed in a retrospective study on pregnant women, which failed to detect any association of alcohol consumption at any level (0.5-14+ drinks *per* week) with increased time to pregnancy, in nulliparous women, although a very modest borderline significant association was found in parous women with high alcohol consumption (14+ drinks *per* week) [132]. Lastly, a prospective cohort study focused on the impact of lifestyle factors on fertility demonstrated that pregnancy rate was not affected by alcohol intake, independently from the level of consumption (less than 5-10+ drinks *per* week) [133]. The two most recent studies investigating the relationship between alcohol consumption and female fertility attempted to determine the relative contribution of different types of alcoholic beverages to the putative risk of infertility. A case-control study [134] nested within the ‘Seguimiento Universidad de Navarra’ (SUN) cohort, a prospective cohort of Spanish university graduates enrolled by the multipurpose SUN project [135], investigated the relationship between self-reported difficulty in getting pregnant and alcohol intake [134]; the study included women reporting difficulty in getting pregnant and women which did not consult due to difficulty in conceiving and had at least one child during follow-up, and reported no association between self-reported difficulty in getting pregnant and alcohol intake (0-5+ drinks *per* week), a result which persisted irrespective to the type of alcoholic beverage [134]. A prospective cohort study enrolled women naïve to fertility treatment and followed until pregnancy or start of fertility treatment, or until the end of observation for a maximum of 12 menstrual cycles; no significant changes were reported in fecundability ratios across alcohol consumption levels (1-14+ drinks *per* week), compared to no alcohol consumption [136]. Moreover, compared with no alcohol intake, the consumption of only wine (≥3 drinks *per* week), only beer (≥ 3 drinks *per* week), or only spirits (≥2 drinks *per* week) did not significantly change fecundability ratios [136]. Lastly, a different cohort study suggested that wine drinkers had a slightly reduced risk of time to pregnancy of more than 12 months, compared to both non-drinkers and consumers of beer or spirits [137]. Moreover, no association was reported between beer drinking and risk of time to pregnancy of more than 12 months, whereas a J-shaped relationship was found for spirits, with a decreased risk in the moderate intake group, and increased risk in the higher intake group [137]. A case-control study in women with a diagnosis of alcoholism and a control group of non-alcohol abuser women with depression, both hospitalized at a psychiatric clinic, reported a non-significant difference in number of pregnancies between the groups [138]. Clinical studies investigating the relationship between alcohol consumption and ART outcomes are scant. A prospective cohort study on women enrolled in ART demonstrated that alcohol consumption during the year before the technique was negatively associated with the number of oocyte retrieved and any documented alcohol consumption during the month preceding the technique tended to increase the risk of not achieving a pregnancy, although this trend was not significant [139]. These findings were confirmed by a cross-sectional study investigating the impact of lifestyle interventions, due to upcoming infertility treatment, in women enrolled in ART; the results of the study demonstrated that women who abstained from drinking or reduced alcohol intake had increased odds of becoming pregnant, compared to women who maintained their habitual alcohol consumption prior to ART [140]. Additionally, a dose-dependent negative effect of alcohol consumption on embryo quality [141, 142], and reduced odds for blastocyst formation [142] were also detected. The discrepancy between findings on the impact of alcohol consumption on natural fertility and assisted reproduction are harsh to address, due to highly heterogeneous study designs, proxy reporting of exposure, and non-standardized assessment and quantification of exposure to alcohol, limitations affecting both types of studies; moreover, misalignment of results may be generated by the existence of extremely scarce investigations in the ART category, probably due to the high motivation in achieving a pregnancy in women enrolled in ART programs, which likely reduced the number of women reporting alcohol consumption prior to ART.

In conclusion, the majority of studies suggest that alcohol consumption might be unrelated to natural fertility, whereas a consistent detrimental effect of alcohol consumption has been reported in ART outcomes.

### Ovarian reserve

Observational and experimental studies in humans and animal models investigating the effects of alcohol consumption on folliculogenesis and oogenesis are scant. Experimental studies on animals are fairly limited, due to the scarcity of suitable models of alcohol consumption; nevertheless, ovotoxicity of alcohol intake has been reported, although the exact mechanisms underlying such an effect, and whether it implies a local action on the ovary or a central action on the HPO axis has still to be completely elucidated. Human studies provided inadequate results and further investigation is still required to definitely clarify whether alcohol consumption has any definite direct effect on the ovary. Scarce observational studies investigated the relationship between alcohol consumption and primordial follicle pool, ovarian reserve, or the risk of POF, and findings have remained inconclusive so far, although either no association [61, 143–147] or a positive association [148–152] between alcohol consumption and older age at menopause has been reported. Alcohol intake was found to be not associated with serum AMH and FSH levels in a large cross-sectional study [47], nor to age-specific AMH percentiles in a large population-based study [42], suggesting that alcohol consumption might not be involved in follicle atresia, nor might affect the number or function of antral follicles, although a different small study showed significantly increased serum FSH levels, and a decreased number of antral follicles, in moderate drinkers compared to non-drinkers [153]. Similarly, a prospective cohort study on women undergoing ART failed to detect any relationship between alcohol consumption and serum AMH levels and small AFC [48], therefore corroborating the assumption of the lack of an effect of alcohol consumption on the antral follicle cohort. More importantly, a cross-sectional study on pre-menopausal women undergoing incidental oophorectomy at the time of hysterectomy, demonstrated that cumulative alcohol consumption, measured as drink-years calculated as average drinks *per* day multiplied by years of alcohol use, was positively associated with ovarian NGF count [46]; in particular, women reporting light (0-1 drink-years) and moderate (1–3 drink-years) alcohol intake had increased NGF counts, whereas women with high (more than 3 drink-years) intake only displayed a trend to an increase, compared to women with no intake of alcohol [46]. Moreover, sensitivity analyses performed by stratifying results based on the recency of alcohol consumption, highlighted that women with both current (consumption within previous year) and past (no consumption within previous year, but positive history of consumption) alcohol consumption had increased NGF counts [46]. These results are in line with those studies reporting delayed menopause in alcohol users, compared to non-users [148–152]. Taken together, these studies might suggest that alcohol consumption at adulthood positively impacts on primordial follicle pool and has no effect on antral follicles; nevertheless, these studies did not account for prenatal alcohol exposure, occurring within an exceptionally sensitive timeframe, coinciding with ovarian development and primordial follicle pool formation, which might represent a strong limitation of studies. A longitudinal, population-based birth cohort study focusing on the impact of in utero exposure to parental alcohol consumption, found that neither maternal nor paternal alcohol intake during pregnancy was related to serum AMH levels in daughters, as assessed at adolescence [60]; these results on trans-generational alcohol exposure, might either suggest that in utero alcohol exposure might have, theoretically, a negligible or no effect on primordial follicle pool development, and, therefore, ovarian reserve markers at adolescence, or, on the other hand, simply confirm the lack of effect of alcohol exposure on antral follicle cohort, as claimed by studies on adult alcohol exposure [42, 47, 48]. Anyhow, the gap between in utero exposures and any assessment performed later in life might represent a source of overlooked confounders: no studies so far coupled in utero exposure to alcohol with a direct measurement of primordial follicle pool; therefore, validation is needed to definitely clarify whether a putative link between alcohol consumption and the status of primordial follicle cohort exists. Lastly, case-control studies investigating pre-menopausal risk factors related to POF failed to find any association between alcohol consumption and the occurrence of POF [61, 62]. Experimental in vivo studies investigating the direct effects of ethanol administration on human ovarian cells are lacking. Nevertheless, one in vitro study on human granulosa cells demonstrated that ethanol treatment had no direct toxic effects, at the tested concentrations, therefore suggesting that granulosa cell viability, a crucial factor which may impact on follicle development, might not be affected by alcohol [154]. Experimental in vivo studies on genetically modified UCh strain rats, an animal model for alcoholism selectively manipulated to generate animals with a differential preference and tolerance to 10% ethanol beverage, and comprising ethanol drinker (UChB) displaying higher, and low-ethanol drinker (UChA) displaying lower tolerance, have been widely used to assess the effects of alcohol on different alcoholism-related diseases; nevertheless, the effects of alcohol consumption on folliculogenesis, oogenesis and ovarian reserve are extremely scarce. One study demonstrated that both UChB and UChA females had a significantly reduced number of primordial follicles, compared to control animals, with UChA females displaying the most severe reduction [155]. Moreover, follicle atresia was found in both UCh strains, although significantly increased atresia was only evidenced in UChB animals, compared to controls, and was markedly detected in antral follicles [155]. Lastly, an experimental in vivo study in a different strain of rats subjected to chronic ethanol administration, suggested that local ethanol metabolism within the ovary increased susceptibility to oxidative stress, which might in turn induce tissue damage, therefore suggesting a local action of ethanol on ovarian structure [156]. Taken together, these results suggest that ethanol affects folliculogenesis and oogenesis, in vivo, nevertheless, dissecting the implication of local actions on the ovary or central actions on the HPO axis requires further investigation.

In conclusion, human studies provided inadequate results to definitely clarify whether alcohol consumption has any effect on ovarian aging and/or ovarian reserve.

### Steroidogenesis

Observational, interventional and experimental studies demonstrated that even moderate alcohol consumption and acute alcohol administration affect the endocrine profile in women of reproductive age, by elevating, and suppressing, estrogens and progesterone levels, respectively; the mechanisms underlying these endocrine effects are still to be completely elucidated; nevertheless, proposed mechanisms include a decreased rate of hepatic estradiol oxidation and an increased aromatization of testosterone to estradiol, a decreased rate of hepatic conversion of pregnenolone to progesterone and reduced uptake of cholesterol, and a dose-dependent and time-dependent decrease in LH/human chorionic gonadotropin (hCG) receptors in granulosa cells, therefore involving both ovarian and extra-ovarian actions of alcohol [157]. Opposite endocrine effects were reported in alcohol abusers, which displayed reduced estrogens and increased progesterone levels compared to non-alcohol abusers. A major role in estrogens-related and progestins-related endocrine derangement induced by alcohol has to be ascribed to the liver, in particular in the case of chronic alcohol consumption, with changes in steroid hormones occurring before liver damage has appeared; nevertheless, in cases of alcoholism determining severe liver damage, disentangle a selected effect of alcohol on estrogens and progestins metabolism from the general effects of liver disease is quite puzzling, therefore dampening the results presented by studies on alcoholism [158]. An observational prospective cohort study attempted to address the relationship between alcohol consumption and estradiol levels, by trying to minimize intra-individual variability and circadian hormone variations by repeating hormone measurement twice, at a one-year interval, within the same phase and on the same day of the menstrual cycle, and at the same hour of the day, by also accounting for other confounders [159]. The results of the study demonstrated that women who consumed alcohol had significantly higher serum levels of total estradiol, compared with abstainers, when estradiol levels were analysed as average of two measurements over a year [159]; moreover, when stratifying women according to alcohol intake categories, estradiol serum levels were found to be higher in the higher-intake categories, suggesting a dose-dependent effect, although differences in estradiol levels among different strata did not reach statistical significance [159]. Observational studies specifically addressing the impact of alcoholism on estrogens and progesterone levels attained inconsistent findings. In particular, two prospective studies conducted in alcohol abusers *vs.* non-alcohol abusers found no significantly different levels of estradiol and estrone, although opposite trends, specifically, higher [160], or lower [161] levels were reported, in alcohol abusers *vs.* non-alcohol abusers; the same studies highlighted that alcoholism did not significantly change progesterone levels, although progesterone levels tended to be higher in the follicular phase of the menstrual cycle [160], and lower in the mid-cycle [160] and luteal phase of the menstrual cycle [160, 161], in alcohol abusers. Conversely, a different case-control study demonstrated that estradiol levels were significantly reduced, and progesterone levels were significantly increased, in alcohol abusers, as compared to non-alcohol abusers [158]. Discrepancies among studies in alcohol abusers might be accounted by differences in the definition of alcoholism, in the amount of alcohol consumed, and in the age of participants. Moreover, none of these studies took into consideration the circadian variations of hormones levels. Lastly, although these studies were performed in alcohol abusers in absence of overt liver damage, a contribution of liver dysfunction on the observed endocrine derangements can’t be completely disregarded. An interventional study on healthy women subjected to acute administration of ethanol solution (0.695 g/kg), prepared with 40% beverage ethanol (vodka), and administered during the follicular phase of the menstrual cycle over a 19 minutes interval, demonstrated that acute administration of alcohol significantly increased plasma estradiol levels within minutes from alcohol administration [162]; these findings were in line with previous reports from similar studies performed during the mid-luteal phase of the menstrual cycle [163], but were in contrast with a previous study on pregnant women with alcohol abuse reporting reduced estradiol levels [164], therefore suggesting a dose-dependent, biphasic, effect of alcohol intake on estrogens, or, again, a contribution of liver dysfunction to decreased estradiol levels in alcohol abusers. The mechanisms beneath the acute response to moderate alcohol ingestion have not been fully addressed in humans, nevertheless, the rapidity of estradiol surge potentially rules out the hypothesis of an effect of alcohol on aromatase activity and testosterone aromatization to estradiol, which has only been demonstrated in a long-term chronic setting of alcohol ingestion, in men [165] and male animal models [166], and indirectly postulated in experimental in vitro studies on human granulosa cells [167]; more likely, a role for the decrease in the liver NAD^+^/NADH ratio caused by increased ethanol hepatic metabolism might be hypothesized, indeed, in these conditions, hepatic estradiol to estrone oxidation is less favored, and might determine estradiol accumulation [157, 162]. Consistently, a different interventional study on healthy women subjected to acute administration of ethanol solution (0.4g/kg), prepared as 8% ethanol in lingonberry juice, demonstrated that intake of alcohol significantly increased and decreased plasma testosterone and androstenedione levels, respectively, reflecting a reduced liver oxidation of androgens [168]. These results corroborate the implication of hepatic steroids metabolism as the mechanisms driving the alcohol-induced increase in estrogens levels; on the other hand, the excess of testosterone might also represent a stimulus to push hepatic aromatase activity toward increased estradiol production, nevertheless, this mechanism has not been validated in interventional or experimental in vivo studies so far. A different interventional study performed across six consecutive menstrual cycles evaluated the effects of moderate alcohol consumption on pooled plasma and urinary estrogens levels, at different time-points within the menstrual cycle, in the attempt to detect differential changes in estrogens levels, according to different phases of the menstrual cycle [169]. Alcohol consumption was set as 30 g of ethanol *per* day, for three consecutive menstrual cycles, with ethanol withdrawal for the last three cycles. The hormonal assessment showed significantly increased plasma levels of estrone and estradiol, and urinary levels of estradiol during the periovulatory phase, whereas, in the luteal phase, a significant increase in urinary levels of estrone, estradiol and estriol were found [169]. These results further strengthen the evidence of a link between alcohol consumption and increased estrogens levels. Moreover, in the specific experimental setting, the lack of changes in the excretion of estrogens catabolites pinpoints to increased estrogens production, rather than variations in estrogens clearance [169]. Conversely, an observational prospective cohort study across a single monitored menstrual cycle, failed to find any association between self-reported alcohol consumption and pooled plasma estrogens levels, in any of the menstrual cycle phases [170]; nevertheless, the pattern of alcohol intake was not accounted for, in this study, in which the median weekly alcohol intake was 51 g [170], therefore, discrepancies between the two studies might be due to different experimental settings. Lastly, the alcohol-induced increase in estrogens levels has been also shown in post-menopausal women, and has been shown to enhance the effects of estrogen replacement therapy and to maintain estrogens levels, after medication removal [157]. Interventional human studies suggested that alcohol ingestion might have adverse effects on progesterone production. A study on women subjected to acute administration of ethanol solution (0.34-1.02 g/kg), demonstrated that intake of alcohol significantly decreased progesterone levels, although no dose-dependent effects were detected [171]. Moreover, two different studies found that alcohol ingestion significantly inhibited progesterone raise during the early follicular phase of the menstrual cycle in women receiving naltrexone [172], and during the luteal phase of the menstrual cycle in women receiving hCG [173]. The hypothesized mechanism driving the observed alcohol-induced progesterone suppression implies a reduced rate of hepatic conversion of pregnenolone to progesterone, an enzymatic process which might be adversely affected by the decreased liver NAD^+^/NADH ratio caused by increased ethanol hepatic metabolism [172]. Experimental in vitro studies on human luteinized granulosa cells confirmed the stimulatory effect of alcohol on estradiol production. In particular, treatment with ethanol at different concentrations induced a dose-dependent increase in basal estradiol secretion by reaching a significant increase at 20 mM [154]; nevertheless, ethanol concentrations above 20 mM did not further increase estradiol secretion by granulosa cells, therefore supporting the hypothesized biphasic action of ethanol observed in vivo. Increased aromatase activity has been indirectly proposed as the driving mechanisms of increased basal estradiol production, since ethanol treatment was shown to significantly increase estradiol levels, in the presence of androstenedione excess [167]. Moreover, at a low concentration of 5 mM, ethanol significantly enhanced FSH-induced estradiol secretion, but this effect was not observed at higher concentrations of ethanol [154]; conversely, ethanol dose-dependently and significantly decreased LH-induced estradiol secretion [154]. The same in vitro study on human luteinized granulosa cells highlighted comparable effects of ethanol treatment on progesterone secretion; in particular, a weak although significant increase in basal progesterone secretion, an inconsistent effect on FSH-induced progesterone secretion, and a significant decrease in LH-induced progesterone secretion [154]. Further in vitro experiments showed that ethanol treatment induced a significant dose-dependent and time-dependent decrease in LH/hCG receptors in granulosa cells, therefore explaining the observed effects of ethanol on LH-induced estradiol and progesterone secretion [154]. These experimental results suggest that ethanol might exert direct and indirect effects on ovarian steroidogenesis by potentially acting at two different levels, namely, increased aromatization of androgens, and decreased LH/hCG receptors expression, therefore stimulating basal estrogens secretion while simultaneously reducing the ovarian response to LH stimulation on estradiol and progesterone synthesis. These results might also address the inhibitory effect of alcohol ingestion on naltrexone-stimulated and hCG-stimulated progesterone production observed in clinical studies. Lastly, an in vitro study on human cytotrophoblast cells isolated from normal-term placenta demonstrated that ethanol treatment dose-dependently decreased progesterone secretion, by possibly blocking the trafficking of cholesterol within cytotrophoblast cells organelles [174]; nevertheless, the exact mechanisms beneath this action are still unclear, and need further investigation.

In conclusion, alcohol consumption has been associated to derangements of the endocrine function, mediated by both ovarian and extra-ovarian actions, although mechanisms underlying the endocrine effects are still to be completely elucidated, in particular in moderate drinkers, displaying higher estrogens and lower progesterone levels, whereas a more complex and opposite profile occurs in alcohol abusers, with a prominent role of non-specific interference of chronic alcoholism, and therefore general liver dysfunction in such endocrine picture.

### Ovulation and menstrual cycle

Observational and experimental studies suggest that alcohol consumption might affect puberty, although a more consistent effect has only been shown in animal models. The specific effects of alcoholism on menstrual cyclicity and ovulation are difficult to address, in the presence of overt alcoholic liver disease, which might independently affect these functions; conversely, observational and experimental studies demonstrated that even moderate alcohol consumption might disrupt menstrual cycling in female humans and animals, by inducing a variety of menstrual cycle disorders, including irregular menstrual cycles and anovulation. The HPO axis has been identified as a main target of alcohol, and hypothalamic dysfunction has been pointed out as a primary site of action, determining decreased LH levels, and driving ovulatory dysfunction and menstrual cycle disorders, including luteal phase insufficiency. Scant investigation has been performed in humans, in mother-daughter couples, addressing the impact of prenatal alcohol exposure on age at menarche; two studies reported that low to moderate alcohol consumption during pregnancy had no effect on the timing of puberty of female descendants [175, 176], a result which was also observed in mothers reporting at least one binge drinking episode, defined as having more than eight drinks in a night or day, during pregnancy [175]. Conversely, an earlier study showed that heavy drinking during pregnancy was related to delayed puberty onset in female daughters, which showed a trend to late menarche [177]. Several observational studies investigated the relationship between alcohol consumption at adulthood and menstrual cycle disorders and menstrual symptoms. Observational studies in alcohol abusers are scarce, and provide no substantial insights into the specific effects of chronic high intake of alcohol on menstrual cycle disorders; indeed, it is known that ovulatory dysfunction and menstrual cycle disorders are associated to chronic liver disease [178], and amenorrhea, anovulation and luteal phase dysfunction appear to be overrepresented in women with alcohol abuse-induced liver damage, due to aberrant estrogens metabolism and HPO axis misalignment [179, 180], therefore, disentangle a specific effect of alcohol consumption from an overall effect of liver disease and general health impairment is quite challenging in studies involving alcohol abusers, unless investigation is replicated in women prior to liver damage. A dated case-control study reported a significantly increased frequency of irregular menstrual cycles in alcohol abusers, although the results were not controlled for relevant confounders including smoke and drug abuse, which significantly differed in the case and control groups [181]. A different case-control study evaluating the impact of active alcoholism on the menstrual pattern demonstrated that menstrual cycle length and menstrual flow were unrelated to the duration of alcohol abuse, and the average menstrual cycle length did not differ, whereas average duration of the menstrual flow was significantly shorter, in alcohol abusers *vs.* non-alcohol abusers [182]. Moreover, greater fluctuations in cycle length and duration of menstrual flow were reported during active alcohol abuse, compared to non-alcohol abusers; in particular, maximal and minimal cycle length were significantly longer and shorter, respectively, and minimal duration of the menstrual flow was significantly shorter, whereas no differences were noted in maximal duration of the menstrual flow [182]. Furthermore, the occurrence of menstrual cycle disorders was significantly more frequent in alcohol abusers *vs.* non-alcohol abusers, and mainly included metrorrhagia, whereas no differences were reported in the frequency of oligomenorrhea, hypermenorrhea, hypomenorrhea, polymenorrhea and polymenorrhagia [182]. Lastly, a different study reported two cases of menses cessation after 3-7 months of heavy drinking, which occurred in absence of significant liver disease [183]. Further studies attempted to determine the mechanisms driving alcohol-induced menstrual cycle disorders in non-cirrhotic alcohol abusers, and reported that serum levels of FSH and LH were similar in the alcohol abusers and non-alcohol abusers throughout the menstrual cycle [160, 161], suggesting that heavy alcohol drinking might have only minor effects on the secretion of gonadotropins, in absence of a frank liver injury, and that the hypogonadotropic hypogonadism observed in the amenorrheic women with alcoholic liver cirrhosis might be more dependent on liver injury than on chronic alcohol consumption as such [160, 161]; nevertheless these results might be interpreted with caution, considering the small sample size of studies. The majority of studies failed to find any association between alcohol consumption and dysmenorrhea [93, 95, 99, 100], although a prospective cohort study reported that consuming alcohol more than once *per* week was associated to an increased probability of having a longer duration of severe pain within the menstrual cycle [97]. A national survey on a representative sample of healthy women investigated the relationship between alcohol consumption and the probability of reporting one or more menstrual cycle disorders and symptoms, among pre-menstrual discomfort, dysmenorrhea and heavy menstrual flow [184]. The results of the study demonstrated that alcohol consumption dose-dependently increased the probability of reporting menstrual cycle problems; moreover, a significant association between episodes of binge drinking and increased reporting of menstrual cycle problems was found [184]. Additionally, a different and more recent study investigating the relationship between the level of alcohol intake over three consecutive weeks of alcohol availability, demonstrated that 50% of women defined as social drinkers (~3.84 drinks *per* day) and 60% of heavy drinkers (~7.81 drinks *per* day) had significant disturbances of the menstrual cycle, compared to occasional drinkers (~1.22 drinks *per* day), and to social drinkers consuming less than 3 drinks *per* day [185]; the most prominent abnormality found in social drinkers was anovulation, which was associated to reduced levels of LH [185]. Interestingly, an interventional study on healthy women administered luteinizing hormone releasing hormone (LHRH) and ethanol solution (0.694 g/kg), demonstrated that alcohol ingestion had no effect on LHRH-stimulated LH secretion, in both the early follicular and mid-luteal phase of the menstrual cycle [186], suggesting an intact hypothalamus-pituitary crosstalk, and that alcohol intake might affect LH secretion from the pituitary by acting upstream to the pituitary; anyhow, these endocrine actions of alcohol might potentially prevent LH surge and ovulation, by leading to luteal phase dysfunction. Nevertheless, the results of a large prospective cohort study did not support the notion that consuming alcohol affects ovulatory function to the point of increasing the frequency of infertility due to ovulation disorders [130], although the lack of an effect on fertility might result as a net balance between delayed menopause and impaired ovulation. Overall, ovulatory and menstrual dysfunction might be more prominent than expected in women with moderate alcohol consumption, and melt off the reliability of findings from studies on alcohol abusers, which seemed to suggest no effect of alcohol on gonadotropins; indeed, although the primary site of action of alcohol within the HPO axis has not been clearly indicated by human studies, experimental in vivo studies in both rats and monkeys demonstrated an alcohol-induced reproductive disruption similar to that seen in humans and provided further insights into the mechanisms beneath the effects of alcohol intake on puberty onset, ovulatory dysfunction and estrous and menstrual cycle disorders, by suggesting a central action of alcohol on the hypothalamic function. Pioneering in vivo studies on prenatally exposed rodents and pre-pubertal rodents demonstrated that chronic ethanol administration caused deferred puberty, as revealed by late vaginal opening and absent or irregular estrous cycles, changes which were accompanied by suppressed serum estrogens, LH, and insulin-like growth factor 1 (IGF-1) levels [187–191]. Studies in pre-pubertal non-human primates similarly reported that chronic ethanol administration affected pubertal hormonal activation; in particular, suppressed serum levels of estrogens and LH were reported, along with suppression of the night-related increase in serum growth hormone (GH) levels, which was expected in late juvenile development, and these changes were paralleled by a decrease in serum IGF-1 levels and irregular monthly pattern of menstruation [192]. Further studies traced back to both LHRH-LH-estrogens and growth hormone releasing hormone (GHRH)-GH-IGF-1 axes as major targets of alcohol [190, 193], and demonstrated that alcohol might exert inhibitory actions indirectly, *via* diminished IGF-1 neuroendocrine signaling, which, in turn, affects in an integrative and bidirectional manner the functionality of both axes, and, directly, at the hypothalamic level, *via* inhibition of the synthesis and release of LHRH, by interference with kisspeptins, and *via* inhibition of GHRH release [190, 193]. An additional role of alcohol in delayed puberty, by means of increased opioid restraint on pubertal progression has also been hypothesized [187]. Besides the effects of alcohol on the complex neuroendocrine and endocrine networking occurring prior to, and orchestrating, the onset of puberty, and consistently with human studies, alcohol exposure at even acute and moderate levels was reported to affect ovulatory function and estrous and menstrual cyclicity in in vivo studies on animal models. Experimental studies in rats demonstrated that acute administration of ethanol at moderate doses or at doses mimicking binge drinking significantly disrupted normal estrous cycling, blocked the proestrous LH surge and ovulation, and significantly reduced serum estrogens and progesterone levels [194–196], although strain differences were noted in the degree of ovulatory dysfunction and normal cyclicity in chronic exposure studies [197]. Additional experiments attempted to determine whether ethanol-induced LH surge suppression was driven by estrogens deprivation and subsequently prevented positive feedback on the hypothalamus, or by direct actions at the hypothalamic-pituitary level [194]; the results of the study demonstrated that acute ethanol administration maintained its inhibitory effects on LH surge in ovariectomized females administered estrogens, therefore disproving the hypothesis of an indirect effect evoked by the lack of estrogens-driven positive feedback, whereas exogenous administration of LHRH to ethanol-treated animals restored LH secretion and ovulation, indicating a central action of ethanol on hypothalamic LHRH synthesis or secretion [194]. Similar results were replicated in studies on non-human primates, which further reported alcohol-induced suppression of basal LH, decreased number and frequency of LH pulses, and delayed or absent LH surge, in estrogens repleted, ovariectomized, monkeys [198, 199]; therefore strongly corroborating the centrality of the hypothalamus-pituitary crosstalk in ovulatory and menstrual dysfunction, in models of alcohol intoxication. These neuroendocrine findings are consistent with menstrual cycle disruptions observed in a primate model of alcoholism [200, 201], and consistently resume aberrations found in alcohol-dependent women [183] and social drinkers [185, 186].

In conclusion, moderate alcohol consumption is suggested to affect the onset of puberty and regular menstrual cycling, and to increase the rate of anovulatory cycles, although the exact mechanisms in humans have not been definitely clarified.

### Oviduct function

Observational and experimental studies addressing the relationship between alcohol consumption and the occurrence of ectopic pregnancy, and the effects of alcohol consumption or intake on oviduct function are extremely scarce, and no clear evidence has been provided so far. A dated case-control study reported that regular alcohol consumption was unlikely to represent a predisposing factor to ectopic pregnancy, based on the similar incidence of alcohol use between women with ectopic pregnancy and a control group comprising women with intra-uterine pregnancies planned to continue to term and intra-uterine pregnancies deliberately interrupted [202]. A more recent large prospective cohort study evaluated the relationship between alcohol consumption and hospitalization for pregnancy outcomes and infertility examinations, by reporting no significant differences in the risk of ectopic pregnancy across alcohol consumption categories, which included non-drinkers plus low consumers, moderate consumers, and high consumers, based on qualitative questionnaires [123]. Lastly, a case-control study in women with a diagnosis of alcoholism and a control group of non-alcohol abusers with depression, both hospitalized at a psychiatric clinic, reported a non-significant difference in number of ectopic pregnancies between the groups [138]. Although no sufficient amount of observational data exists to definitely establish whether alcohol consumption might be linked to the occurrence of ectopic pregnancy, experimental studies in humans and animal models suggested that alcohol intake during early pregnancy affects oviduct function and embryo transport. An experimental study on human fallopian tubes obtained from salpingectomy for ectopic pregnancy and hysterectomy or postpartum sterilization samples demonstrated that in vitro treatment with ethanol significantly and reversibly inhibited frequency and amplitude of spontaneous contractions of both ampulla and isthmus smooth muscle, an effect which was accompanied by a significant increase in NO synthase expression and in NO content; these effects were attenuated in the presence of a NO synthase competitive inhibitor [203]. Moreover, in vivo acute (74 hours) exposure experiments on female mice selected for the presence of vaginal plugs, and treated with ethanol (4g/kg/day) at different time-points after vaginal plug detection, demonstrated that acute intake of ethanol at early pregnancy stage significantly delayed embryo transport within the oviduct and early embryo development, as demonstrated by the recovery of a larger number of embryos at the 2-cell, 4-cell and morula stages at day 4 of pregnancy, and by the presence of asynchronous development [203]; these aberrations were mostly rescued by co-treatment with a NO synthase competitive inhibitor, therefore suggesting that NO pathway and inhibition of oviductal smooth muscle contractions might have mediated ethanol-induced defects [203]. In a similar setting, a chronic (30 days) exposure to ethanol (4g/kg/day) determined the destruction of the oviductal epithelium in the isthmus region, whereas the epithelium lining of the ampulla region was disorganized, with loss of ciliated epithelial cells and breakdown of ciliary structure [203], therefore suggesting that, other than smooth muscle, ciliated epithelium might be a target of ethanol toxicity within the oviduct.

In conclusion, alcohol consumption seems unrelated to ectopic pregnancy, although experimental studies in humans suggest that it might affect oviductal smooth muscle contraction.

### Uterus receptivity and implantation

Chronic alcohol consumption during pregnancy is known to be associated with severe detrimental effects on the fetus [3, 122]; nevertheless, the effects of alcohol consumption around peri-conceptional and pre-implantation period potentially affecting development of the embryo prior to placental development and/or implantation itself or uterus receptivity is unclear. Human studies specifically addressing this issue are lacking, mostly due to difficulties in reporting precisely the amount of alcohol consumed during this definite period; however, mechanistic insights are provided by in vitro studies. In vivo studies in animal models trying to address the effects of ethanol on uterine physiology and pre-implantation events have been unsatisfactory so far, since administration methods itself elicited responses which might independently affect outcomes, therefore acting as potential confounders, which included ethanol-induced reduction of caloric intake leading to undernutrition, and stress response *via* activation of glucocorticoid pathway [204]. Alcohol is able to enter into uterine lumen, where it may directly affect the developing pre-implantation embryo [205]. Noteworthy, several experimental in vitro studies on pre-implantation mouse and porcine embryos highlighted dose-dependent biphasic effects of alcohol, with low doses shown to speed-up pre-implantation morphogenesis, mediated by calcium-induced enhanced cavitation, cell proliferation, blastocyst outgrowth and precocious differentiation [204], and, as opposite, high doses shown to disturb embryo development, as evidenced by increased apoptosis, reduced total cell number, and reduced blastocyst formation and hatching [204]. Moreover, direct effects of ethanol have also been examined in cultured human cytotrophoblasts, which demonstrated reduced cell proliferation [206], likely determined by induction of apoptosis [207, 208]. Lastly, experimental studies in human undifferentiated embryonic stem cells treated with ethanol demonstrated DNA hypermethylation, with downregulation of stem cell maintenance markers including Oct4, Nanog and Sox2 [209]; nevertheless, whether ethanol might pass into the blastocoel and reach the embryonic stem cells in vivo has not been determined so far.

In conclusion, studies on alcohol consumption and uterus receptiveness and/or implantation in humans are scarce, although experimental in vitro studies suggest that alcohol might exert detrimental effects on cytotrophoblast development and pre-implantation events.

## Drug addiction and female fertility

Drug addiction represents a serious concern, being associated to a wide range of short-term and long-term, direct and indirect effects on human health, depending on the specific drug or drugs, route of administration, and frequency of use or abuse [210]. Despite the well characterized effects on other domains of human health, and the evidence that the consumption of addictive drugs during pregnancy poses real risks to the developing fetus and neonate, the effects on female reproductive function and fertility of drug addiction, and, even less, of drug use at lower levels, have been scantly investigated in clinical observational studies [3, 210], the majority of studies focusing on marijuana, which is the most commonly abused drug worldwide, whose psychoactive component is Δ^9^-tetrahydrocannabinol (Δ^9^-THC) [211], whereas other addictive drugs have been disregarded. Moreover, an explicit association between selected addictive drugs and specific reproductive effects is severely dampened by the predominance of multi-drug consumption, by the heterogeneity in levels and patterns of use, and by the presence of confounding factors represented by the general health consequences of addiction, which include multiple debilitating stressors, overall unhealthy lifestyle, and poor decision making, which might independently affect reproductive outcomes and fertility. Lastly, some studies have suggested that addictive women are also more prone to attitudes which might represent a confounder in the assessment of fertility potential and pregnancy rate, such as risky sexual behaviours, more frequent intercourses, and adolescent pregnancy [212, 213]. Most of the evidence on the reproductive effects of addictive drugs has been retrieved from experimental studies in animal models, which were mostly focused on marijuana.

### Marijuana

Few observational studies assessed the effects of marijuana on ovulatory function and menstrual cycle in humans, and reported controversial results, due to heterogeneity in study design, selection of women with occasional or chronic marijuana use, lack of control for the amount of marijuana at each dose and/or for the simultaneous use of different drugs. A population-based case-control study on women with ovulatory dysfunction and women with tubal infertility, and age-matched fertile women, demonstrated that marijuana significantly increased the risk of ovulatory infertility [214], although a different population-based case-control study on women with any cause of primary infertility surprisingly demonstrated that the average time to conception was significantly shorter for women who had used marijuana regularly [131]. A prospective cohort study investigating the effects of marijuana on menstrual cycle disorders reported an association between occasional marijuana use and longer follicular phase of the menstrual cycle, resulting in delayed ovulation, and a slightly elevated rate of anovulatory cycles [215]. The study also reported no effect of marijuana on luteal phase length [215]. Conversely, a different study demonstrated that chronic marijuana use was associated to an increased percentage of menstrual cycles with a shorter luteal phase and, in line with different studies, to an increased rate of anovulatory cycles [216]. Studies on the effects of marijuana on the HPO axis are scarce; the results of these studies demonstrated that an acute inhaled dose of marijuana suppressed serum LH levels during the luteal, but not follicular phase of the menstrual cycle [217, 218]. Interestingly, a significant increase of serum LH levels was reported following marijuana inhalation during the periovulatory phase [219]. Experimental studies in animal models, mostly rhesus monkeys, subjected to acute, sub-chronic and chronic dosing of Δ^9^-THC, clearly identified HPO axis disruption as the mechanisms underlying the disturbance of ovulatory function and menstrual cycle by marijuana. An in vivo study in rhesus monkeys demonstrated that a single injection of Δ^9^-THC during the mid-luteal phase of the menstrual cycle decreased serum progesterone levels, an effect which was reversed by the administration of hCG [220], suggesting that the effect of Δ^9^-THC was mediated by suppression of gonadotropin release from the pituitary, rather than a local effect on the ovary. This assumption was consistent with previous findings from an in vivo study on ovariectomized rhesus monkeys, which demonstrated that a single injection of Δ^9^-THC decreased serum levels of both LH and FSH [221]. Studies with sub-chronic Δ^9^-THC administration demonstrated a differential susceptibility of menstrual cycle phases to the effects of Δ^9^-THC; specifically, daily injections throughout the luteal phase of the menstrual cycle failed to affect serum progesterone levels, and to affect luteal phase length [222], whereas a similar treatment regimen throughout the follicular phase was shown to increase menstrual cycle length, to decrease estrogen and progesterone serum levels, to disrupt follicular development, and to inhibit LH surge and prevent ovulation, therefore increasing the rate of anovulatory cycles, effects which were reversed by mid-cycle gonadotropin administration [223]. Studies with chronic Δ^9^-THC administration by 3 injections *per* week throughout several consecutive menstrual cycles strongly decreased serum levels of estradiol, progesterone and LH, and blocked ovulation and menses; nevertheless, animals developed tolerance to Δ^9^-THC, and these effects were overcame with time [224]. Additional experimental studies aimed at further characterizing the endocrine effects of Δ^9^-THC provided robust evidence that the hypothalamus, rather than the pituitary, is the specific target within the HPO axis responsible for dropping of serum gonadotropins levels upon treatment; indeed, Δ^9^-THC-induced decrease of gonadotropins levels was efficiently recovered by the administration of exogenous gonadotropin releasing hormone (GnRH) [221], suggesting that the pituitary maintained responsiveness to hypothalamic hormones in the presence of Δ^9^-THC. Lastly, Δ^9^-THC failed to suppress GnRH secretion in vitro [225], therefore, modulation of neuronal stimuli inhibiting GnRH release, rather that inhibition of GnRH release into the pituitary portal vasculature has been proposed as the mechanism of Δ^9^-THC action at the hypothalamic level [226, 227].

In conclusion, marijuana seems to induce disturbance of ovulatory function and menstrual cycle, leading to increased ovulatory infertility rate, effects which are proposed to be mediated by central actions at the hypothalamic level, as suggested by in vitro studies and studies in animal models.

### Other addictive drugs

Few observational studies and experimental studies in animals have been performed, concerning the relationship between cocaine and female fertility, whereas only one observational study has been performed on heroin addicts, and the effects of methamphetamine have been scantly investigated in experimental studies in animal models.

A population-based case-control study on women with ovulatory dysfunction and women with tubal infertility, and age-matched fertile women demonstrated that cocaine significantly increased the risk of primary tubal infertility [214]. A different and larger population-based case-control study on fertile women and women with any cause of primary infertility demonstrated that cocaine did not modify the risk for infertility, and that the average time to conception was significantly shorter and the risk of conceiving was significantly increased (as assessed restrictively in live-born pregnancies), for women who had ever used cocaine than for women who had never used cocaine [131]. Although reporting opposite results, both studies had inherent limitations linked to the retrospective design, which included recall biases, lack of information concerning the quantity and frequency of cocaine use, and the recency of cocaine use relative to conception; therefore, a dose-dependent relationship could not be evaluated, and further studies are required to draw definitive conclusions. Experimental in vivo studies in rats demonstrated that chronic treatment with subcutaneous injections of 1-20 mg/kg/day cocaine induced a dose-dependent impairment of estrous cyclicity, and that, at 20 mg/kg cocaine, estrous cycle disruption was permanent, even after discontinuation of treatment [228]; moreover, serum LH levels and ovulation rate were significantly reduced by treatment with 10 and 20 mg/kg/day cocaine, whereas cocaine had no effect on serum levels of FSH [228]. These results were confirmed by in vivo studies in rhesus monkeys, which reported significantly impaired menstrual cyclicity and increased rate of anovulation, upon chronic cocaine self-administration sessions; moreover, over 25% of menstrual cycles remained anovulatory during cocaine withdrawal [229]. The mechanism underlying the observed alterations of estrous and menstrual cyclicity and decrease in LH levels might include either direct inhibition of LH release from the pituitary, or an indirect action on ovarian hormones, and subsequent impairment of the feedback loop on the pituitary. Nevertheless, in vivo studies on rhesus monkeys demonstrated that acute treatment with intravenous injections of cocaine significantly increased LH levels [230], and significantly enhanced exogenous LHRH-induced stimulation of LH secretion from the pituitary [231], therefore suggesting that cocaine does not directly suppress the hypothalamus-pituitary crosstalk. Conversely, one in vivo study on rats injected intraperitoneally with 15 mg/kg cocaine, thrice a day, for one day, demonstrated that cocaine significantly increased serum progesterone levels, although progesterone returned to baseline after three hours [232], suggesting that this change was an acute effect of cocaine administration. Nevertheless, a different in vivo study on rats investigating the reciprocal interactions among ovarian hormones, hypothalamus-pituitary-adrenal axis, and responses to cocaine, demonstrated that a single intraperitoneal injection of 15 mg/kg cocaine increased progesterone secretion in sham-adrenalectomized, but not in adrenalectomized rats, pinpointing to an extra-ovarian source of cocaine-induced progesterone secretion [233], and suggesting that adrenally-derived progesterone might contribute to cocaine-induced inhibition of LH release by negative feedback mechanisms. Lastly, an in vivo study in mice subjected to chronic treatment with 40 mg/kg cocaine administered by 2 subcutaneous daily injections, demonstrated that treated animals had significantly delayed puberty, and were impregnated at older ages than controls, but cocaine treatment had no discernible effects on their offspring [234]. Only one very dated study evaluated the effects of heroin addiction on female fertility, by retrieving records on women addicts from mixed sources, including women included in previous studies assessing different endpoints, women seeking for assistance at late pregnancy, and prisoners incarcerated during pregnancy and attending clinics for heroin withdrawal and delivery. The results of the study demonstrated that amenorrhea invariably occurred in heroin addicts with an addiction history of 1 to 4 months, whereas menstrual cyclicity was restored upon withdrawal [235]. One experimental in vivo study on rats treated by daily subcutaneous injections of 5 mg/kg methamphetamine, for approximately nine weeks, starting prior to pregnancy and throughout lactation, demonstrated that there was a significant difference in the regularity of the estrous cycle in methamphetamine-treated animals, compared to controls, with methamphetamine-treated females cycling irregularly in 100% cases, although no differences were found in the incidence of successful pregnancy and litter size [236]; nevertheless, gestation was significantly longer in methamphetamine-treated animals, and a trend toward a lower incidence of successful delivery was found [236]. Conversely, a different in vivo study on mice treated by intraperitoneal injections of 5 mg/kg methamphetamine, once a day for three consecutive days *per* week, from the 21^st^ postnatal day for eight consecutive weeks, demonstrated that there was no significant difference in the regularity of the estrous cycle, between methamphetamine-treated and untreated animals [237]. Nevertheless, the results of the study demonstrated that methamphetamine significantly reduced primordial follicles pool and the percentage of growing follicles, and increased follicle atresia [237], changes which were accompanied by reduced expression of AMH, and increased or decreased expression of pro- and anti-apoptotic factors, respectively, within the ovary [237]. Lastly, in vitro secretion of AMH, estradiol and progesterone was significantly reduced, in ovarian granulosa cells isolated from the methamphetamine-treated mice, although no obvious morphological abnormalities were detected [237].

In conclusion, the most consistent effect of cocaine is an increase in progesterone and a decrease in LH levels, which are suggested to be driven by extra-ovarian production of progesterone, namely, secretion by the adrenal gland, which in turn exerts negative feedback on LH secretion, in in vivo studies in animals. These hormonal changes are likely connected to the impaired estrous and menstrual cyclicity and increased rate of anovulation observed in treated animals. Experimental in vitro studies on ovarian granulosa cells isolated from treated animals demonstrate that methamphetamine reduces both estradiol and progesterone, suggesting a direct effect on ovarian steroidogenesis. No data are available on the effects of heroin on steroidogenesis, however, amenorrhea invariably occurs in heroin addicts, although menstrual cyclicity is recovered upon withdrawal.

## PCOS and endometriosis

The majority of observational studies addressing the relationship between smoking and PCOS features, demonstrated that smoking further increased androgens levels in PCOS patients [238, 239], a result which is largely consistent with the endocrine profile displayed by healthy non-PCOS smokers, although biochemical hyperandrogenism was not reflected by clinical hyperandrogenism [238, 239], whereas no effect was reported on different clinical signs of PCOS, including acne, menstrual disorders and oligo-anovulation [238, 239], neither on polycystic ovary morphology or different ultrasound parameters [238, 239]. Studies on in utero exposure to parental smoke or alcohol consistently demonstrated lack of an association between maternal smoking [240–242] or alcohol consumption [243, 244] and the risk of developing endometriosis in the female descendants; interestingly, one study demonstrated a linear positive association between indoor exposure to passive smoking during childhood and the risk of endometriosis [245]. Metanalyses of studies on patients with clinically and/or histologically diagnosed endometriosis provided no evidence of an association between smoking at adulthood and the risk of endometriosis, irrespective to smoking status, amount of cigarettes smoked, and type of endometriosis and study design [246], whereas an increased risk of endometriosis in women reporting current, but not former, alcohol consumption at adulthood was described, in fertile but not infertile women [247]. Moreover, alcohol consumption was found to be associated to an increased risk of deep endometriotic nodules, but not peritoneal endometriosis [247].

## Conclusions

Despite mounting interest on the impact of preventable lifestyle-related factors on female reproductive function, studies addressing the effects of smoke, alcohol and addictive drugs are still controversial, and inherent limitations rule out the possibility to establish definitive inference of causality. The majority of studies suggest that natural fertility is decreased in current smokers and prenatally smoke-exposed women, whereas alcohol consumption seems to be unrelated to natural fertility, and sparse and inconclusive data exist concerning addictive drugs. Moreover, relatively scant studies report a controversial relationship between smoking and ART outcomes and a more consistent detrimental effect of alcohol consumption. Insufficient or no experimental investigation has been performed so far, therefore, although targeted actions have been reported, further studies are needed to definitely identify the specific affected domains of the female reproductive function, and to clearly depict the underlying mechanisms of reproductive toxicity. Observational studies suggest that smoking might affect ovarian reserve markers, although the relationship between smoking and POF is controversial; experimental studies in humans suggest that an impairment of antral follicle development and growth due to supportive granulosa cells-directed toxicity, rather than primordial follicle pool depletion, might mediate these effects. Observational, interventional and experimental studies in humans demonstrate that smoke and moderate alcohol consumption are associated to significant derangements of the female endocrine profile, mediated by ovarian and extra-ovarian actions; in particular, smokers are characterized by lower estrogens and progesterone and higher androgens levels, whereas moderate drinkers display higher estrogens and lower progesterone levels. Moreover, despite an increased risk of oligomenorrhea and menstrual symptoms, smoking seems not to be associated to ovulatory dysfunction, whereas moderate alcohol consumption is suggested to affect the onset of puberty and regular menstrual cycling, and to increase the rate of anovulatory cycles. Studies on alcohol abusers report inconsistent findings, due to heterogeneous definition of alcoholism and non-specific interference of liver dysfunction on steroidogenesis, menstrual disorders and ovulation. Observational studies highlight that smoking, but not alcohol consumption, is associated to significantly higher odds for ectopic pregnancy, although experimental studies in humans suggest that both might affect oviductal smooth muscle contraction; moreover, smoking affects both endometrium receptivity and cytotrophoblast proliferation, migration and invasion, therefore determining delayed uterine implantation, whereas evidences of a direct effects of ethanol administration on cytotrophoblast are only provided by few experimental studies. Observational studies on the effects of addictive drugs on steroidogenesis and ovulatory function are sparse and fragmented. The most consistent effect of marijuana is a menstrual cycle phase-dependent decrease of progesterone and LH levels, and an increased rate of anovulatory cycles, which might explain the observed association between marijuana and ovulatory infertility. As opposite, the most consistent effect of cocaine is an increase in progesterone and a decrease in LH levels. No data are available on the effects of heroin on steroidogenesis; nevertheless, amenorrhea invariably occurs in addicted women, although menstrual cyclicity is recovered upon heroin withdrawal.

Lastly, studies specifically addressing the relationship between smoking and two major causes of infertility, namely, PCOS and endometriosis, suggest that, except for exacerbated biochemical, but not clinical, hyperandrogenism, smoking might not affect clinical signs or ovarian morphology, in PCOS patients; moreover, smoking seems to be unrelated to the risk of endometriosis, independently from the timing of exposure or amount of cigarettes smoked, although passive smoking during childhood might increase the risk. Conversely, alcohol consumption is associated to an increased risk of developing endometriosis, with relevant differences concerning current *vs*. former alcohol consumption, fertility status, and differential diagnosis of deep endometriotic nodules *vs.* peritoneal disease. Taken together, available data suggest that each of the described, preventable, unhealthy habits, might affect some domains of the female reproductive function, although these aberrations are not precisely reflected by definite effects on fertility, in some cases, due to heterogeneity or scarcity of investigation; considering that more questions than answers still characterize the effects of smoke, alcohol and addictive drugs on female fertility, relinquishing of these modifiable risk factors is robustly warranted.

## Data Availability

Literature search results are available from the authors on reasonable request.

## Abbreviations

ACTH: Adrenocorticotropic hormone
AFC: Antral follicle count
AMH: Anti-müllerian hormone
ART: Assisted reproductive technologies
FSH: Follicle stimulating hormone
GH: Growth hormone
GHRH: Growth hormone releasing hormone
GnRH: Gonadotropin releasing hormone
hCG: Human chorionic gonadotropin
HPO: Hypothalamus-pituitary-ovary
LH: Luteinizing hormone
LHRH: Luteinizing hormone releasing hormone
NGF: Non-growing follicle
NO: Nitric oxide
PCOS: Polycystic ovary syndrome
POF: Premature ovarian failure
SHBG: Sex hormone binding globulin
Δ^9^-THC: Δ^9^-tetrahydrocannabinol
